# The atypical cadherin flamingo determines the competence of neurons for activity-dependent fine-scale topography

**DOI:** 10.1186/s13041-019-0531-7

**Published:** 2019-12-10

**Authors:** Ruonan Li, Yuhua Liang, Siyang Zheng, Qun He, Limin Yang

**Affiliations:** 1 School of Medicine, Dalian University, Dalian, 116622 Liaoning China; 2 Institute of Health Sciences, China Medical University, Shenyang, 110112 China; 3 Life Sciences Institute, Dalian National University, Dalian, 116600 Liaoning China; 4 Chronic Disease Research Center, Dalian Key Laboratory, Dalian, 116622 Liaoning China; 5 School of Medicine, Jiamusi University, Jiamusi, 154007 Heilongjiang China

**Keywords:** Fine-scale topography, Flamingo, Neuronal activity, Competence, *Drosophila*, Cell-adhesion molecule

## Abstract

The topographic projection of afferent terminals into two-dimensional maps is essential for sensory systems to encode the locations of sensory stimuli. In vertebrates, guidance cues are critical for establishing a coarse topographic map, while neuronal activity directs fine-scale topography between adjacent afferent terminals. However, the molecular mechanism underlying activity-dependent fine-scale topography is not well known. Studies in the *Drosophila* visual system have demonstrated that cell-adhesion molecules direct fine-scale topography, but whether or not these molecules are involved in activity-dependent fine-scale topography remains to be determined. We previously reported that the nociceptors in *Drosophila* larvae form an activity-dependent fine-scale topographic system. The establishment of this system is instructed by the level of neuronal activity in individual nociceptors. Here, we show that the atypical cadherin Flamingo (Fmi) is required for establishing the nociceptor topographic map. We found that the topographic defect caused by loss of *fmi* was epistatic to the inhibition of neuronal activity and the overexpression of the activity-regulated gene *Trim9*. These results suggest that Fmi and neuronal activity interact to regulate fine-scale topography. This study provides a link between neuronal activity and the cell-adhesion molecule in the establishment of fine-scale topography.

## Introduction

Adjacent somatosensory neurons of the same kind form fine projection maps—termed fine-scale topographic maps in the central nervous system (CNS) [1]. In vertebrates, the guidance cues are critical for establishing a coarse topographic map, and neuronal activity then directs fine-scale topography between adjacent afferent terminals [2]. In the visual system of *Drosophila*, many studies have shown that cell-adhesion molecules play a vital role in the formation of fine-scale topography [3–6]. By using genetic mosaic techniques, such as flip-out and mosaic analysis with a repressible cell marker (MARCM) [7], it was found that the cell-adhesion molecules Flamingo (Fmi) and N-cadherin (Ncad) are responsible for axon-axon interactions, which in turn regulate the formation of fine-scale topography [3, 4]. The functions of Fmi and Ncad are different in establishing the topography of photoreceptor axons [5, 6]. Fmi is mainly expressed in the photoreceptors, while Ncad is expressed in both photoreceptors and their targets [3, 4]. Fmi mediates opposing interactions between photoreceptors, while Ncad mediates attractive interactions between photoreceptors and their targets [5, 8]. Whether or not neuronal activity and cell-adhesion molecules interact to establish fine-scale topography is unknown.

The formation of fine-scale topography in the *Drosophila* visual system is independent of neural activity [9], precluding the use of this system for testing the interaction between neuronal activity and cell-adhesion molecules. We previously found that the axon terminals of *Drosophila larvae* nociceptors could form an activity-dependent fine-scale topographic map in ventral never cord (VNC) [10]. Thus, this system offers the opportunity for studying the interaction between neuronal activity and cell-adhesion molecules in the formation of fine-scale topography. In *Drosophila larvae*, the dendrites of the three nociceptors-- termed Class IV dendritic arborization (C4da) neurons --in each hemi-segment partition the body wall of the hemi-segment. We previously found that the axon terminals of the three C4da neurons, ddaC (dorsal, D), v’ada (middle, M) and vdaB (ventral, V), form a fine-scale topographic map in the ventral nerve cord (VNC), which is *Drosophila*’s equivalent of the vertebrate spinal cord [10]. The positions of the axon terminals in the VNC correspond to the dendritic coverages on the body wall, such that the axon of the D neuron projects to the dorsal side of the C4da neuropil in the VNC, the M neuron projects to the middle, and the V neuron projects to the ventral side. Moreover, we showed that the topographic separation of axon terminals between the M and V neurons depends on neuronal activity. Specifically, inhibition of neuronal activity mainly affects M axon terminals and increases activity primarily affects V axon terminals. Neuronal activity regulates the expression of the cytoplasmic protein Tripartite motif protein, Anomalies in sensory axon patterning (Asap), which is also called *Drosophila* Trim9 (*d*Trim9). *d*Trim9 expression level is higher in V neurons than in M neurons [11]. Inhibition of neuronal excitability increases *d*Trim9 levels in M neurons, while increased excitability reduces *d*Trim9 levels in V neurons [10].

In this study, we used the C4da topographic system to test the potential contribution of Fmi to activity-dependent fine-scale topography. We found that the instructive role of neuronal activity and *d*Trim9 in the formation of C4da topographic map disappear upon the loss of *fmi*, suggesting that Fmi determines the competence of neurons for activity-dependent fine-scale topography. This study provides mechanistic insights into the molecular mechanism underlying activity-dependent fine-scale topography.

## Materials and methods

### Fly stocks

The following published fly strains were used: *fmi*^*E59*^ [12], UAS-Fmi [12, 13], *asap*^*91*^(a null allele of *d*Trim9) [11], UAS-3HA-asap [11], UAS-kir2.1 [14, 15], UAS-ork^ΔNC^, UAS-ork^ΔC^ [15]. Flies are reared on standard cornmeal-based food at 25 °C.

### Mosaic analysis for topography

MARCM experiments were employed as previously described [7, 10]. Briefly, we used a C4da specific GAL4 driver, *ppk*-GAL4 [16], to express mCD8-GFP in single C4da neurons through MARCM. A transgene expressing the red fluorescent protein tdTomato directly driven by the C4da-specific *ppk* promoter (*ppk-tdTomato*) [16] is integrated into the MARCM system. The synaptic terminals of single C4da neurons were thus identified within the C4da neuropils. Three hours after egg-laying, the eggs were heat-shocked at 37.5 °C for 15~25 min. The eggs were kept at 25 °C for 72 h after heat shock, and then dissected for immunostaining. Only earlier third instar larvae were dissected to ensure the consistency of the developmental stages of the analyzed animals.

### Immunohistochemistry

Earlier third instar larval fillets were stained with the following primary antibodies: chicken anti-GFP (1:5000, Abcam), mouse anti-GFP (1:5000, Life Technologies), and rabbit anti-RFP (1:5000, Life Technologies). The following secondary antibodies were used: donkey anti-chicken Alexa Fluro® 488(1:500, Jackson ImmunoResearch Inc), donkey anti-mouse Alexa Fluro® 488 (1:500, Jackson ImmunoResearch Inc) and donkey anti-rabbit Rhodamine RX (1:500, Jackson ImmunoResearch Inc).

### Image preprocessing and analysis

The image preprocessing was performed as described [10]. All images were collected as 3D stacks using an FV1000 confocal system (Olympus Microsystems) equipped with a 60× oil lens (Plan-Apochromat, numerical aperture [NA] =1.4, Olympus Microsystems). The z-step was 0.3 μm. Minimum signal saturation when collecting all images and all image stacks were deconvolved with Huygens software (Scientific Volum Imaging). The 3D image analysis software Amira (FEI Visualization Sciences Group) was employed to align all of the VNC in each stack to uniform orientation. The 3D image stacks contain only the C4da neuropil with the single MARCM clones were cropped by software ImageJ (National Institutes of Health). After the above preprocessing, the image stacks were analyzed by custom-designed software for the topographic index (TI) [10]. The software measures the relative position of each clone between the dorsal and ventral boundaries of a C4da neuropil and calculates the TI of each clone. The final TI for each clone was then calculated as the average of TI = ∑TI*i* / n, where n is the total number of cloned voxels in the 3D image stack. The TI analysis was done by the software automatically.

### Statistical analysis

All data are presented as mean values ± SEM. The data were analyzed with Prism GraphPad 6.01. The student’s *t-test* performed a data comparison between the two groups. One-way ANOVA with Sidák correction was used for comparing three or more groups. *P* < 0.05 was considered significant.

## Results

### Flamingo is required for establishing the fine-scale topography of C4da neurons

**Fig. 1:**
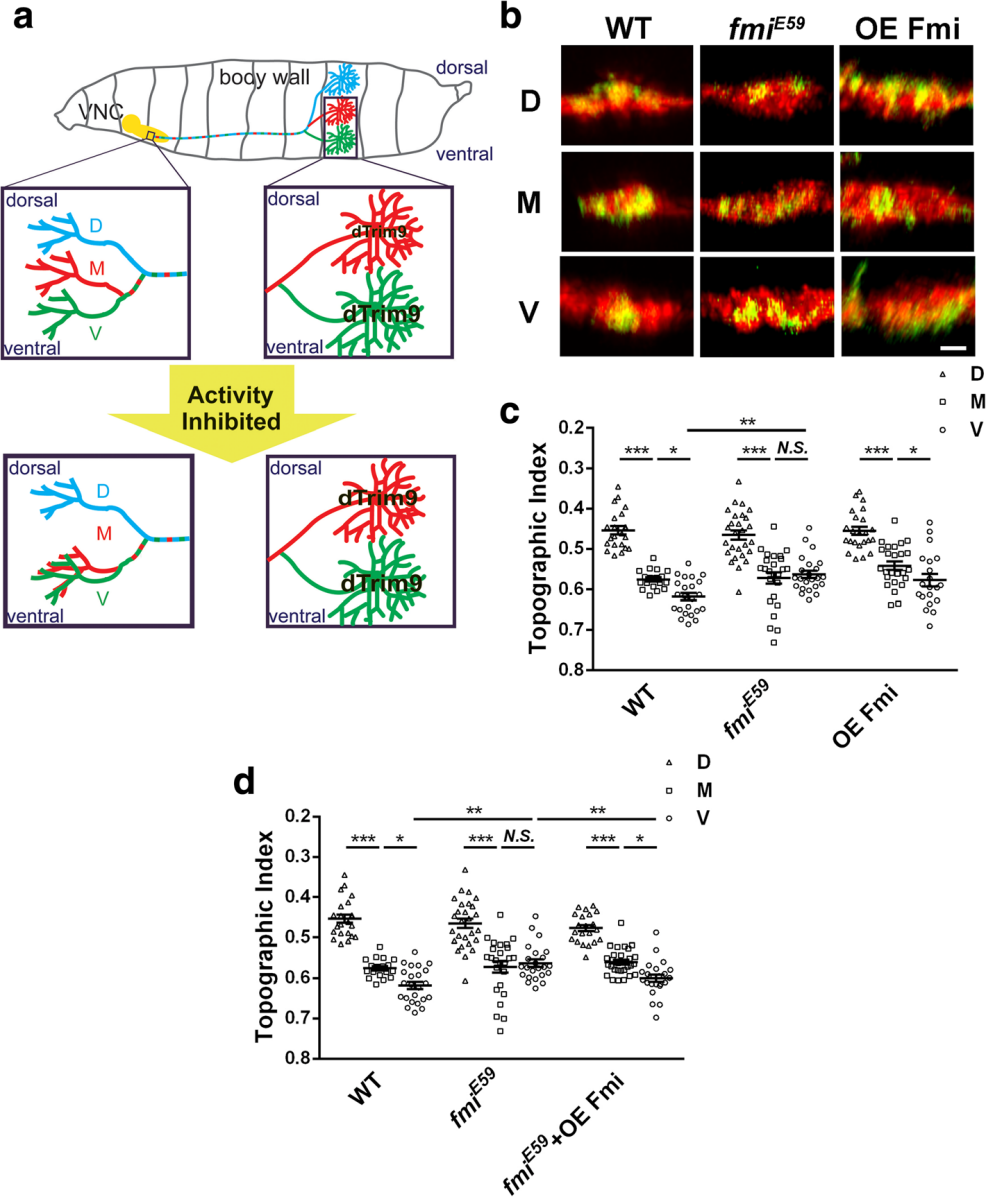
Flamingo is required for the formation of the C4da topographic map. **a** The three C4da neurons (D, M and V) in one hemi-segment of *Drosophila* larvae project into VNC to form an activity-dependent topographic map. This topography depends on the relative levels of *d*Tim9 in these three neurons. The activity inhibition of M neurons shifts their axon terminals ventrally by increasing *d*Trim9 expression. **b** Representative images of the dorsal-ventral view of single C4da terminals (green) and the C4da neuropil marked by *ppk*-tdTomato (red). WT, wild type; *fmi*^*E59*^*,fmi* null allele of mutation; OE Fmi, overexpress flamingo. **c** The statistic analysis of the TI of C4da axon terminals. *Fmi* null mutation causes the axon terminals of V neurons to project toward the dorsal side of VNC, resulting in the mixing of M terminals with V terminals. There is a significant difference in the projection of the V-terminals of the *fmi* mutation and the wild type V-terminals in VNC. Overexpress Fmi has no effects on the formation of topography. **d** Overexpress Fmi rescues the topographic defect in *fmi* mutant C4da neurons. The axon terminals of *fmi* mutatnt V neurons relocated to the ventral side of C4da neuropil by overexpressing Fmi on it. There is a significant difference in the projection of V-terminals of the *fmi* mutation and the *fmi* mutation + OE Fmi. D, ddaC; M, v’ada; V, vdaB. *P < 0.05; **P < 0.01; ***P < 0.001; Scale bar, 2 μm. Error bars indicate mean ± SEM. WT: D, *n* = 22; M, *n* = 18; V, *n* = 23; *fmi*^*E59*^: D, *n* = 27; M, *n* = 25; V, n = 23; OE Fmi: D, n = 23; M, n = 25; V, *n* = 20; *fmi*^*E59*^ + OE Fmi: D, *n* = 21; M,*n* = 29; V, n = 23

The axon terminals of the three nociceptors in each larval hemisegment form an expanded structure in the VNC that is enriched with synapses, which is termed the C4da neuropil [17]. The axon terminals of D, M, and V nociceptors are positioned in the dorsal, middle, and ventral parts of the C4da neuropil, forming a fine-topographic map [10]. The C4da topographic map is established gradually during the development. First, at the embryonic stage, the D axon was separated from the M and V axons by projecting dorsally and entered the C4da neuropil through the dorsal bundle. Second, the terminals of M and V neurons are separated at the early second-instar stage through a neuronal-activity-dependent process (Fig. 1a).

Prompted by previous findings that the cell adhesion molecule Fmi regulates fine-scale topography in *Drosophila* visual system by mediates axon-axon interaction, we set out to determine whether Fmi plays a role in the fine-scale topography in the C4da system. Using the single-cell genetic manipulation technique MARCM, we found that loss of *fmi* in single V neurons shifted the presynaptic terminals of these neurons dorsally to the middle portion of the C4da neuropil. The positions of presynaptic terminals of *fmi−/−* D and M neurons remained indistinguishable from those of wild-type D and M neurons (Fig. 1b, c). Overexpression of Fmi in single C4da neurons did not affect the topographic map (Fig. 1b, c), but completely rescued the topographic defect in *fmi−/−* V neurons (Fig. 1d). These results suggested that the topographic arrangement of the M and V terminals requires *fmi*.

**Fig. 2:**
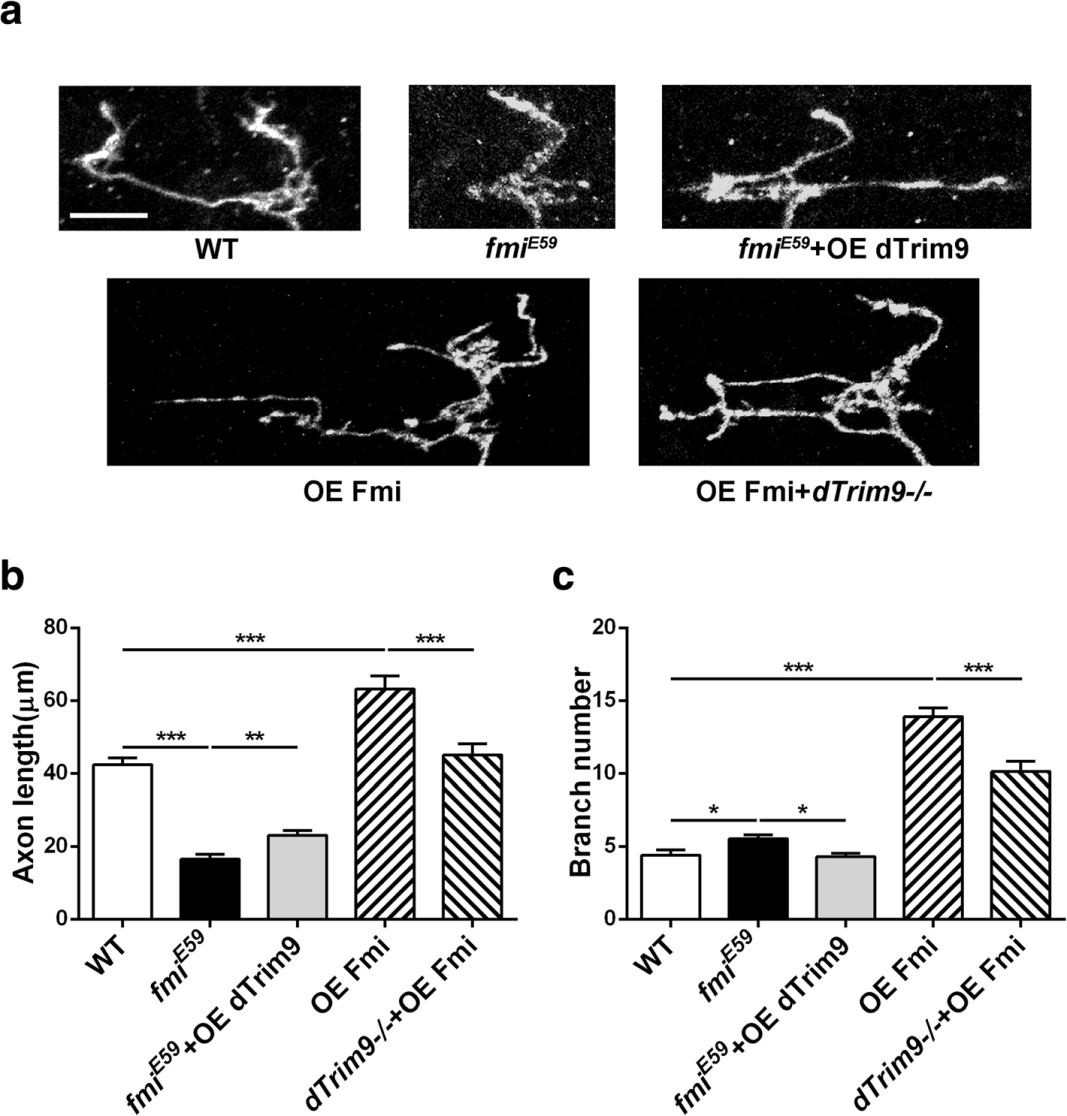
Effects of Fmi on the growth of C4da axon terminals. **a** Images of V axon terminals. **b**, **c** *fmi* mutations lead to shortening of axon terminals and an increase in the number of branches of V neurons. Overexpression of Fmi leads to excessive growth of axon terminals and an increase in the number of branches. *dTrim9* partially rescues the phenotypes caused by *fmi* dysfunction. *P < 0.05; **P < 0.01; ***P < 0.001; WT: n = 22; *fmi*^*E59*^: n = 20; *fmi*^*E59*^ + OE dTrim9: *n* = 19; OE Fmi: n = 20; *dTrim9−/−* + OE Fmi: n = 18

In addition to the topographic defect, loss of *fmi* also reduced the length of C4da axon terminals, whereas overexpression of Fmi led to an overgrowth of these terminals (Fig. 2). These results suggest that Fmi instructs the growth of C4da presynaptic terminals. Fmi’s function in presynaptic growth seems to be distinct from that in topography because the only loss of *fmi*, but not overexpression, affected the C4da topography.

### Flamingo determines the competence of C4da neurons for Trim9-instructed topography

**Fig. 3:**
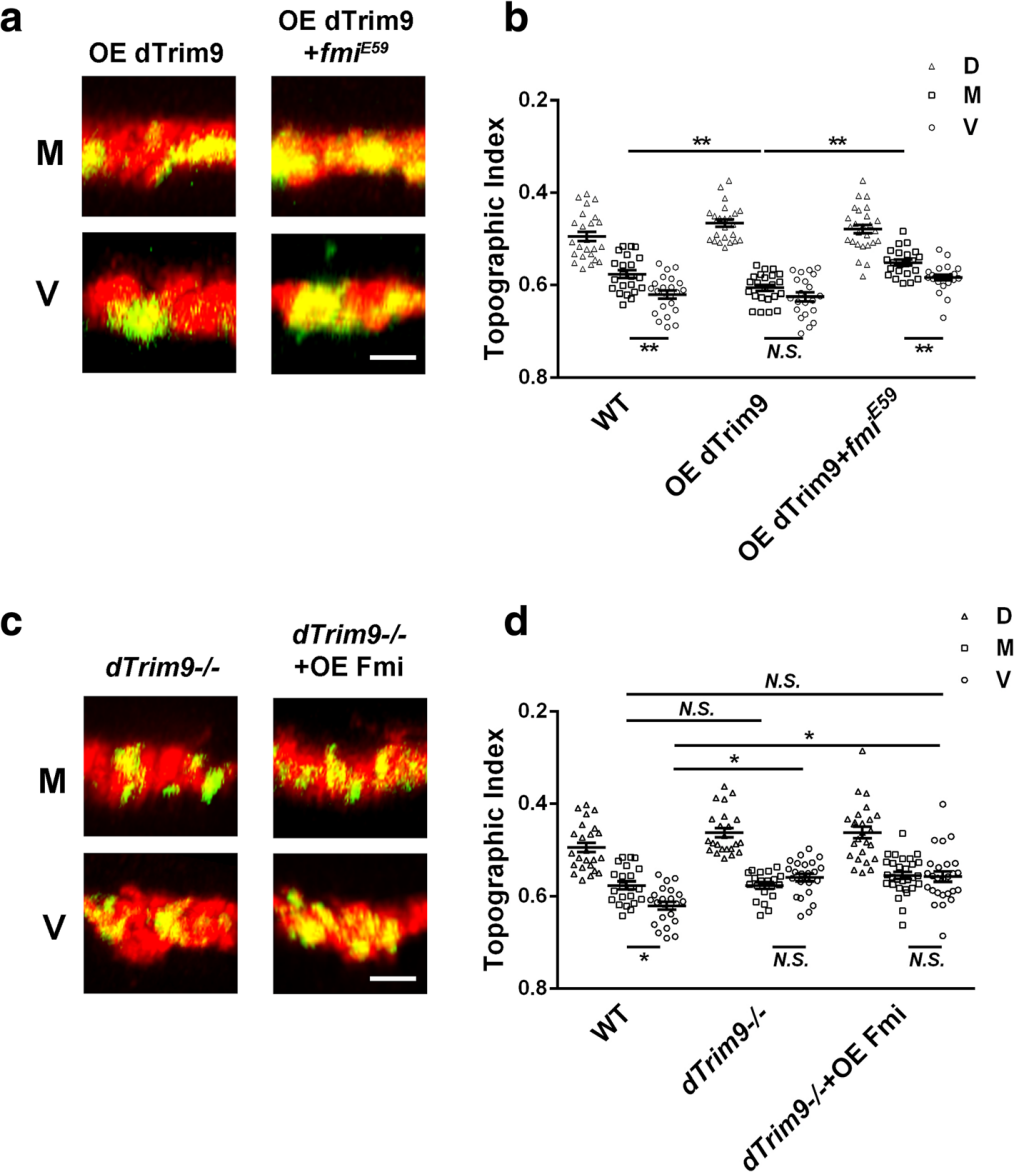
Genetic interactions between Fmi and *d*Trim9 in C4da topography. **a**, **c** Representative images of the dorsal-ventral view of single C4da terminals (green) and the C4da neuropil marked by *ppk*-tdTomato (red). **b** Overexpression of dTrim9 causes the axon terminals of M neurons to shift to the ventral side, mixing with the axon terminals of V neurons, resulting in a defect in topography, but this phenotype disappears in the *fmi* mutant. **c** The statistic results of axon terminals TI of three neurons of C4da. Overexpression of Fmi has no effects on the phenotype of the *dTrim9* mutation. OE, overexpress; −/−, null allele of mutation; D, ddaC; M, v’ada; V, vdaB. *P < 0.05; **P < 0.01; *N.S.*, no significant difference. Scale bar, 2 μm. Error bars indicate mean ± SEM. WT: D, *n* = 24; M, *n* = 22; V, *n* = 23; OE dTrim9: D, n = 24; M, n = 23; V, n = 21; OE dTrim9 + *fmi*^*E59*^: D, *n* = 26; M, n = 22; V, n = 23; *dTrim9−/−*: D, n = 23; M, *n* = 20; V, n = 23; *dTrim9−/−* + OE Fmi: D, n = 25; M,n = 29; V, n = 26

The *d*Trim9 instructs the establishment of C4da topography [10]. The topographic defect caused by loss of *fmi* in single C4da neurons was similar to that caused by loss of *dTrim9* and opposite to *d*Trim9 overexpression. Thus, we decided to test whether *dTrim9* and *fmi* genetically interact with each other to determine fine-scale topography. Epistasis tests were performed to determine whether the topographic effects of *d*Trim9 requires Fmi function. Using the MARCM technique, we overexpressed *d*Trim9 in either wild-type or *fmi−/−* C4da neurons. Wild-type M neurons overexpressing *d*Trim9 projected their presynaptic terminals to the ventral portion of the C4da neuropil [10] (Fig. 3a, b). This topographic defect was entirely blocked by the loss of *fmi* (Fig. 3a, b). By contrast, overexpressing Fmi in *dTrim9−/−* neurons did not change the topographic phenotype of *dTrim9−/−*, which is a dorsal shift of the presynaptic terminals of V neurons (Fig. 3c, d). These results suggested that the capability of *d*Trim9 in regulating C4da topography requires Fmi. In other words, Fmi confers C4da neurons the competence for *d*Trim9-instructed topography.

In contrast to the genetic interactions in topography, overexpressing Fmi in *dTrim9−/−* neurons partially rescued the length and branch numbers of axon terminals (Fig. 2). Moreover, while *fmi−/−* completely eliminated of the topographic defects caused by *d*Trim9 overexpression, it only partially blocked the axon morphological defects caused by *d*Trim9 overexpression (Fig. 2). These results again suggest that the morphology and fine-scale topography of C4da axon terminals are regulated by different mechanisms.

### Flamingo determines the competence of C4da neurons for activity-dependent topography

**Fig. 4:**
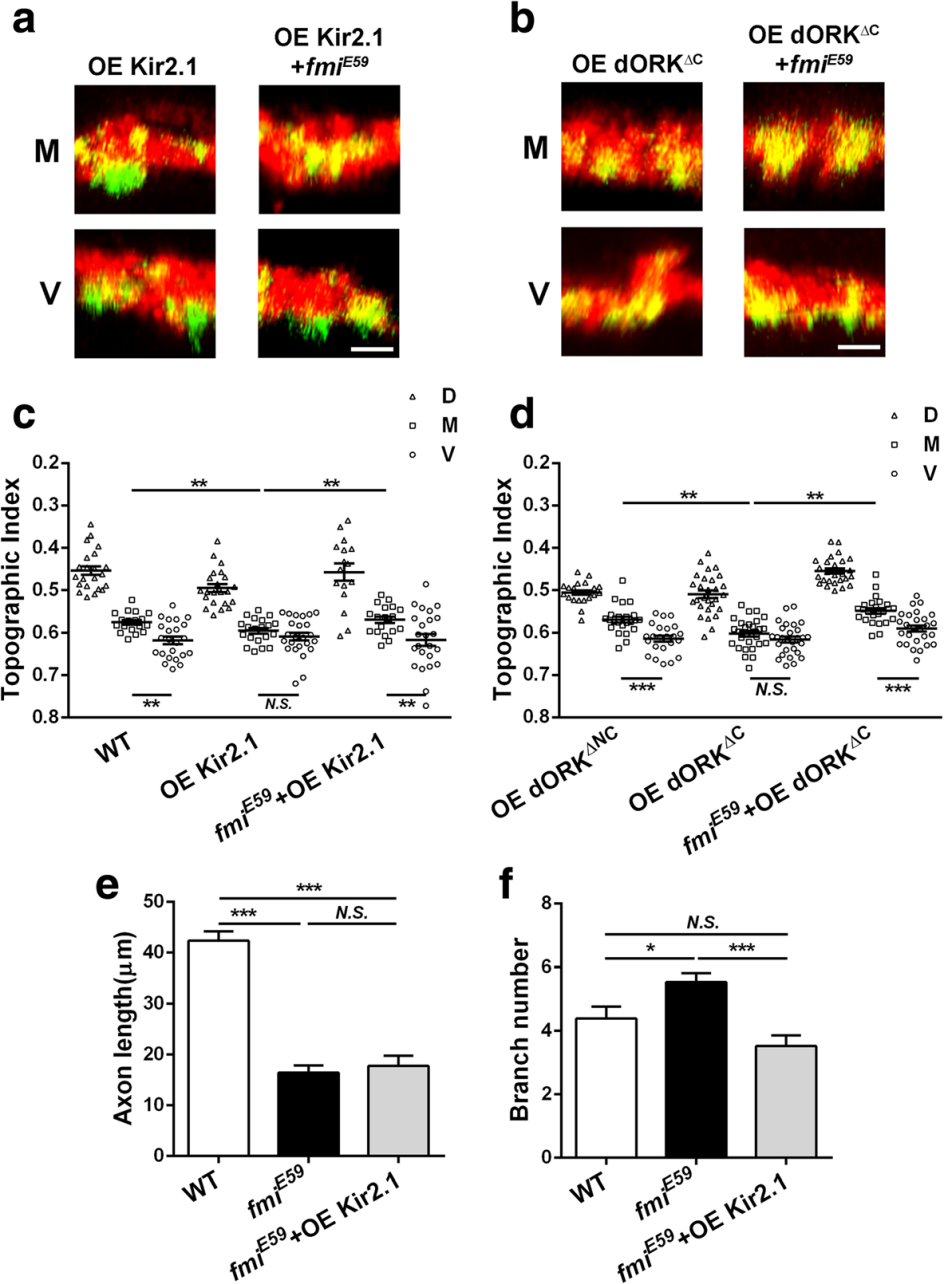
Neuronal activity requires Fmi to instruct C4da topography. **a**, **b** Representative images of the dorsal-ventral view of single C4da terminals (green) and the C4da neuropil marked by *ppk*-tdTomato (red). **c**, **d** are statistic results of the axon terminals index of three neurons of C4da. **a**, **c** Inhibition of neuronal activity by overexpressing Kir2.1. **b**, **d** Inhibition of the neuronal activity of C4da neurons by overexpressing ORK^△NC^ as WT and ORK^△C^. Both **c**, **d** showed that in the *fmi* mutant neurons, the regulation of neuronal activity on topography disappeared. **c** WT: D, n = 22; M, *n* = 18; V, n = 23; OE Kir2.1: D, n = 23; M, n = 20; V, n = 24; *fmi*^*E59*^ + OE Kir2.1: D, *n* = 16; M, *n* = 19; V, *n* = 24. **d** WT: D, n = 22; M, n = 23; V, n = 23; OE ORK^△C^: D, *n* = 28; M, *n* = 30; V, n = 28; *fmi*^*E59*^ + OE ORK^△C^: D, n = 26; M, n = 24; V, *n* = 27. **e** Inhibition of neuronal activity does not affect the axonal shortening caused by loss of *fmi*. **f** Inhibition of neuronal activity can rescue the increased branch’s number of axon terminals caused by *fmi* mutations. D, ddaC; M, v’ada; V, vdaB. *P < 0.05; **P < 0.01; ***P < 0.001; *N.S.*, no significant difference. Scale bar, 2 μm. Error bars indicate mean ± SEM. WT: n = 22; OE Kir2.1: n = 20; *fmi*^*E59*^ + OE Kir2.1: *n* = 17

The separation of the presynaptic terminals of M and V neurons is activity-dependent. Neural activities of M and V neurons regulate *d*Trim9 levels to control the topographic positions of the M and V neurons [10]. The inhibition of neuronal activity results in a topographic defect that is similar to *d*Trim9 overexpression (Fig. 1a). Since *d*Trim9 requires Fmi in instructing topographic projection, we asked whether neuronal activities of C4da neurons also require Fmi to instruct C4da topography. Because inhibiting neuronal activity resulted in a topographic defect that was opposite to *fmi* loss of function, we performed a genetic epistasis test to detect whether the neuronal activity requires *fmi* to regulate C4da topography. Using the MARCM technique, we overexpressed the inward rectifier potassium channel Kir2.1 [18] in *fmi−/−* C4da neurons to robustly inhibit the activity of them. The presynaptic terminals of *fmi−/−* M neurons that overexpressed Kir2.1 shifted dorsally to the middle position (Fig. 4a, c), demonstrating that the topographic phenotype caused by Kir2.1 overexpression requires *fmi*. Replacing Kir2.1 with a constitutively open mutant of *Drosophila* rectifier potassium channel 1 (*d*ORK^△C^) [18] had similar effects (Fig. 4b, d), confirming the results of Kir2.1 overexpression.

Taken together, these results show that, just like *d*Trim9 overexpression, the effect of inhibiting neuronal activity on C4da topography requires Fmi. They suggest that Fmi determines the competence of M and V terminals for topographic separation. Unlike overexpression of *d*Trim9, overexpressing Kir2.1 reduced the number of axon terminal branches in *fmi* mutant neurons (Fig. 4f), but did not affect the length of axon terminals (Fig. 4e). This may suggest that neuronal activity and *d*Trim9 regulate axon terminal morphology through different pathways.

## Discussion

In this study, we examined the role of Fmi in the formation of C4da fine-scale topographic map in *Drosophila* larvae. We found that Fmi is required for the neuronal activity and *d*Trim9 to regulate the formation of fine-scale topography. We also show an instructive role of Fmi in the growth of C4da axon terminals, which appears to be a distinct function from topography.

By using single-cell gene manipulation technique MARCM, we found that loss of *fmi* function leads to the disappearance of the topographic separation of the presynaptic terminals of M neurons and V neurons by shifting V terminals dorsally. Overexpression of Fmi did not affect the C4da topography (Fig. 1c), suggesting that Fmi plays a permissive, but not an instructive role, in establishing C4da topography. This is different from the mechanism of action of Fmi in the *Drosophila* visual system. In the *Drosophila* visual system, both loss and overexpression of Fmi can lead to defects in photoreceptor R1-R6 target selection, and the effect is non-autonomous [5]. Although we did not observe the phenotype of wild-type C4da axon terminals that are surrounded by *fmi−/−* C4da neurons, we speculate that Fmi’s function in C4da neurons is cell-autonomous, because the MARCM experiments showed that *fmi−/−* lead defects of M and V projections. This suggests that the mechanism of action of Fmi in the visual system is different from that in the C4da neurons.

We previously showed that C4da topography is activity-dependent and that neuronal activity determines the expression of *d*Trim9 protein to instruct the formation of C4da fine-scale topography [10] (Fig. 1a). It is different from the formation of visual fine-scale topography, which is independent of the neural activity [9]. Whether it is activity-dependent or non-activity-dependent fine-scale topography, their formations are closely related to the establishment and maintenance of synapses between pre- and postsynaptic neurons. Studies have shown that neurons require the cell adhesion molecule Fmi for synaptic target selection, synaptogenesis, the survival of axons and synapses [13, 19]. We speculate that in the formation of C4da fine-scale topography, the activity-*d*Trim9 signaling pathway instructs the axon of M and V neurons to the target area in C4da neuropil, and then Fmi helps to form synapse between these neurons and their postsynaptic targets. When Fmi is absent, C4da axon terminals fail to stabilize in the target area and do not form correct synapses. Bao et al. showed that *fmi* mutation causes a significant increase in the number of ectopic synapses on muscles [13]. In the C4da system, loss of *fmi* may cause the axon terminals of V neuron to form ectopic synapses, resulting in a dorsal shift of these axon terminals to the middle of C4da neuropil. Supporting this idea, we found that loss of *fmi* led to an increase in the C4da axon terminal branch number, which may reflect ectopic synapse formation. Overexpression of *d*Trim9 and inhibition of neuronal activity reduced the increased branch number caused by *fmi* mutations (Figs. 2c and 4f). To verify this hypothesis, further experiments are needed to observe the changes in synapse distribution of the axon terminals in C4da neurons directly, which is technically challenging at this stage.

In summary, neuronal activity plays an instructive role in the formation of C4da topography, but this role requires the presence of Fmi. Fmi determines the competence of neurons for activity-dependent fine-scale topography.

## Data Availability

The datasets generated and/or analyzed during the current study are available from the corresponding author on reasonable request.

## Abbreviations

C4da neurons: Class IV dendritic arborization neurons
CNS: central nervous system
Fmi: Flamingo
MARCM: mosaic analysis with a repressible cell marker
Ncad: N-cadherin
TI: topographic index
VNC: ventral never cord
